# Comparison of copper concentration between non-alcoholic fatty liver disease patients and normal individuals: A meta-analysis

**DOI:** 10.3389/fpubh.2023.1095916

**Published:** 2023-02-01

**Authors:** Yanfang Chen, Chutian Wu, Guanhong Li, Wen Wang, Shaohui Tang

**Affiliations:** ^1^Department of Gastroenterology, The First Affiliated Hospital, Jinan University, Guangzhou, Guangdong, China; ^2^Department of Gastroenterology, Guangdong General Hospital's Nanhai Hospital, Foshan, China

**Keywords:** non-alcoholic fatty liver disease, copper, serum, hepatic, meta-analysis

## Abstract

Non-alcoholic fatty liver disease (NAFLD) is the most prevalent chronic liver disease worldwide. Copper metabolism plays an important role in the pathogenesis of NAFLD. However, the relationship between serum/hepatic copper concentration and NAFLD is still debated. A literature search was performed using electronic databases to find publications up to September 2022, where the relationship between serum/hepatic copper or ceruloplasmin concentration and NAFLD was evaluated. Finally, 6 articles with 9 unique outcomes involving 2,607 NAFLD patients and 1,441 non-NAFLD normal individuals were included. The pooled results showed that hepatic copper concentration was significantly decreased in NAFLD patients (SMD = −0.98, 95% CI = [−1.21; −0.74], *p* < 0.0001), and the sensitivity analysis also confirmed this. Nevertheless, serum copper (SMD = −0.02, 95% CI = [−0.32; 0.28], *p* = 0.88) and ceruloplasmin (SMD = −0.03, 95% CI = [−0.69; 0.63], *p* = 0.93) were not associated with NAFLD. This meta-analysis revealed that low hepatic copper concentration was found in NAFLD patients and serum copper and ceruloplasmin were not associated with NAFLD. Larger cohort studies and related trials are needed to further validate the result of this meta-analysis in the future.

## Introduction

Non-alcoholic fatty liver disease (NAFLD) is a multistage disease affecting 30% of the global population, which will supposedly emerge as the most common cause of the end-stage liver disease (1, 2). Substantial studies have demonstrated that NAFLD contributes to several disease processes containing hepatic/extrahepatic diseases and an overall increase in mortality (3). Currently, it is well recognized that the double-hit theory plays a vital role in the pathogenesis of NAFLD (4). However, no medicine is consented yet for this condition by the US Food and Drug Administration or the European Medicines Agency (5).

Serum copper is mainly transported by binding to ceruloplasmin which regulates the distribution and release of copper and later played its biological roles (6). Copper is an indispensable trace element that serves as a structural and enzymatic cofactor for various antioxidant proteins, including cytochrome c oxidase (COX), superoxide dismutase (SOD), and ceruloplasmin (7). Excessive or deficient copper can lead to mitochondrial dysfunction or dyslipidemia (8). A typical example is Wilson's disease, an overloaded hepatic copper accumulation and insufficient ceruloplasmin with liver steatosis, inflammation, and cuprotosis (9). However, recent studies demonstrate the association between copper deficiency and the accumulation of fat in the liver, and NAFLD patients with hepatic copper deficiency show more severe liver steatosis, inflammation, and clinical symptoms (10, 11). Besides, rats fed with a restricted copper diet will spontaneously develop liver steatosis (12). These results indicate that copper plays an essential role in the pathogenesis of NAFLD.

Whereas, the relationship between serum/hepatic copper levels and NAFLD is still unclear. What's more, it is reported that lower hepatic copper is associated with NAFLD, but excessive copper also impairs hepatocytes (8, 13). Lan, et al. find that blood copper concentration is lower in NAFLD patients (14). Chen, et al. and Wang, et al. indicate that serum copper level is higher in NAFLD patients compared with normal individuals (15, 16).

Given that the role of copper in NAFLD is still controversial, this study performed a meta-analysis of the studies on serum/hepatic copper or ceruloplasmin levels to clarify their relationship.

## Materials and methods

### Data sources and search strategy

We searched Pubmed, EMBASE, Cochrane Library, Web of Science, and China National Knowledge Internet (CNKI) databases from inception to September 20, 2022. Key search terms included: (“NAFLD” or “fatty liver”) AND (“copper” or “cuprum” or “ceruloplasmin”). We also manually screened the citation of selected articles to identify the eligible articles. The present systematic review was conducted in accordance with the Preferred Reporting Items for Systematic Reviews and Meta-Analyses (PRISMA) checklist, and the protocol had been registered at PROSPERO (www.crd.york.ac.uk/PROSPERO, ID: CRD42022361187).

### Study selection

**Figure 1:**
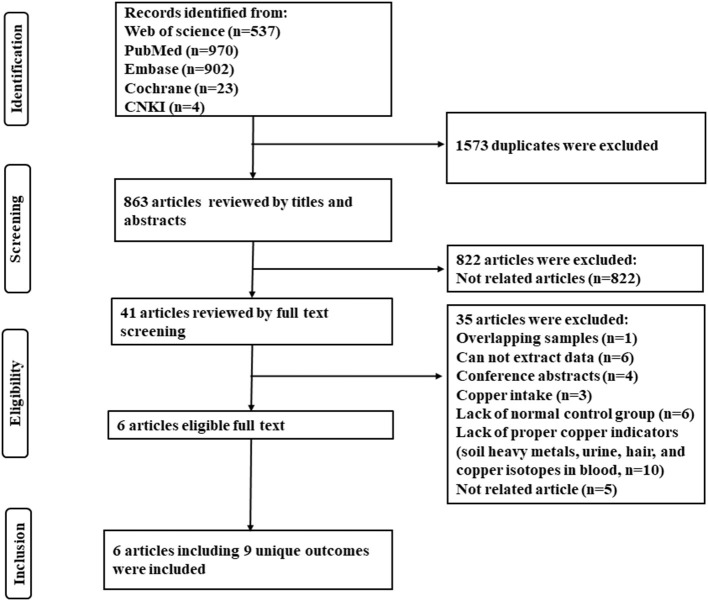
The PRISMA flow diagram of the study selection process.

Abstracts and titles were reviewed to filter out irrelevant studies. Potentially relevant studies had their full text extracted and two reviewers (Guanhong Li and Wen Wang) independently assessed articles for inclusion/exclusion criteria. Dissonance was resolved by discussion. Inclusion criteria included: (1) studies comparing serum copper or hepatic copper levels between NAFLD patients and non-NAFLD normal individuals (control group); (2) diagnosis of hepatic steatosis by imaging or biopsy; (3) full-text articles available. Exclusion criteria included: (1) studies using overlapping samples; (2) those did not contain non-NAFLD normal control; (3) researches about copper intake; (3) incomplete or unextractable raw data; (4) reviews, letters, case reports, editorials, conference abstracts, and animal experiments. A summary of studies identified, screening, eligibility, and exclusion is shown in the PRISMA flow diagram (Figure 1).

### Data extraction and quality assessment

Two reviewers (YF and CT) independently performed data extraction and solved dissonance by discussion. Data extracted for eligible studies included the first author, publication year, study location, study design, the diagnostics method of NAFLD, serum/hepatic copper levels or ceruloplasmin and standard deviation (SD), sample size, the method for the detection of copper, age, and sex.

Newcastle-Ottawa Quality Assessment Scale (NOS) was used to assess the methodological quality of included studies. Risk of bias was categorized as follows: 7–9 was regarded as high-quality, 4–6 as moderate-quality, and under 4 as low-quality (17).

### Statistical analysis

All statistical analyses were performed using “meta” R package in R software (Version 4.2.1, www.r-project.org). Heterogeneity was evaluated using the χ^2^ test, tau^2^ test, and Higgins I^2^ test. If *p* value ≥ 0.05 and I^2^ ≤ 50%, suggesting little homogeneity among the studies, the fixed effect model was used for analysis. If *p* value < 0.05 and I^2^ > 50%, indicating substantial heterogeneity among different studies, the random effects model was used for analysis. If there was heterogeneity among the studies, subgroup analysis was conducted to detect the source of heterogeneity. Standardized mean differences (SMD) and a 95% confidence interval (CI) of serum/copper levels were calculated for continuous variables. A sensitivity analysis was performed by omitting one study and pooling the SMD for the others in each turn. Publication biases were determined by Egger's test. A *p* value < 0.05 was considered statistically significant.

## Results

### Study selection and characteristics

As shown in Figure 1, 2,436 articles were included in the initial research, and 6 articles with 9 unique outcomes were finally retrieved after filtration. The 9 researches were published from 2008 to 2022, involving 2,607 NAFLD patients and 1,441 non-NAFLD normal individuals (control group). One study was cross-sectional and 5 studies were case-control. Two studies were carried out in China, 2 in Austria, 1 in America, and 1 in Iran. The NOS scores shown that 3 studies were rated as high quality and 3 were considered as moderate quality (Table 1).

**Table 1:** The characteristics of included study.

							**NAFLD**		**CNTR**		

**Author**	**Country**	**year**	**Copper detection**	**Diagnostic method**	**Type**	**Study design**	**Age**	**Male/Female**	**Age**	**Male/Female**	**NOS**
Arefhosseini. et al. (10)	Iran	2022	Commercial kits	US	SCu	Cross-sectional	37.07 ± 11.78	18/67	26.48 ± 13.19	19/37	6
Lan. et al. (14)	China	2021	ICP-MS	US	SCu	Case-control	56.3 ± 12.12	970/846	56.4 ± 12.3	598/513	9
Wang. et al. (16)	China	2011	FAAS	US	SCu	Case–control	39.13 ± 10.69	105/45	38.94 ± 10.66	105/45	7
Aigner. et al. (18)	Austria	2008	Laboratory methods	Biopsy	SCu	Case–control	52.4 ± 11.1	84/56	43.6 ± 9.3	8/16	6
Arefhosseini. et al. (10)	Iran	2022	Commercial kits	US	SCer	Cross-sectional	37.07 ± 11.78	18/67	26.48 ± 13.19	19/37	6
Mendoza. et al. (19)	America	2017	NA	Biopsy	SCer	Case–control	13.9 ± 3	77/25	13.4 ± 5	16/32	7
Mendoza. et al. (19)	America	2017	NA	Biopsy	HCu	Case–control	13.9 ± 3	77/25	13.4 ± 5	16/32	7
Stattermayer. et al. (13)	Austria	2017	FAAS	Biopsy	HCu	Case–control	49.1 ± 3	120/54	34.1	14/12	6
Aigner. et al. (18)	Austria	2008	MS	Biopsy	HCu	Case–control	52.4 ± 11.1	84/56	43.6 ± 9.3	8/16	6

The data were shown as mean ± standard deviation (SD). NAFLD, non-alcoholic fatty liver disease; CNTR, control group; ICP-MS, inductively coupled plasma mass spectrometry; FAAS, flame atomic absorption spectroscopy; MS, mass spectrometry; NA, not available; US, ultrasound; NOS, Newcastle-Ottawa Quality Assessment Scale; SCu, serum copper; SCer, serum ceruloplasmin; HCu, hepatic copper.

### Overall meta-analysis

**Figure 2:**
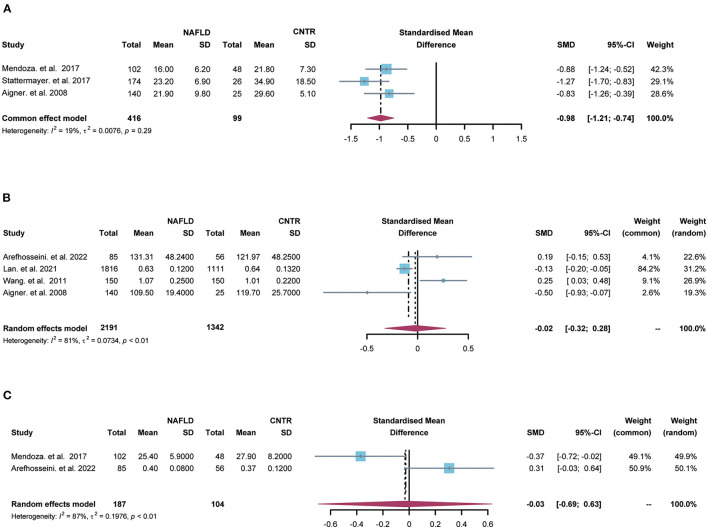
The forest plots for comparisons of hepatic copper **(A)**, serum copper **(B)** and ceruloplasmin **(C)** between NAFLD and the control group. SD, standard deviation; SMD, standardized mean differences; CI, confidence interval.

Meta-analysis was performed to compare the hepatic/serum copper or ceruloplasmin levels between NAFLD patients and the control group. There were 3 researches about hepatic copper and the heterogeneity among the studies was little (I^2^ = 19%, tau^2^ = 0.0076, *p* = 0.29). The hepatic copper level was significantly decreased in NALFD patients (SMD = −0.98, 95% CI = [−1.21; −0.74], *p* < 0.0001, Figure 2A) using the fixed effects model.

Moreover, 4 studies contained serum copper levels and substantial heterogeneity was found among the studies (I^2^ = 81%, tau^2^ = 0.0734, *p* < 0.01). There was no significant relationship between serum copper and NAFLD (SMD = −0.02, 95% CI = [−0.32; 0.28], *p* = 0.88, Figure 2B) using the random effects model. Besides, 2 studies included serum ceruloplasmin and large heterogeneity was found among the studies (I^2^ = 87%, tau^2^ = 0.1976, *p* < 0.01). There was no significant relationship between serum ceruloplasmin and NAFLD (SMD = −0.03, 95% CI = [−0.69; 0.63], *p* = 0.93, Figure 2C) by the random effects model.

### Sensitivity analysis and publication bias

**Figure 3:**
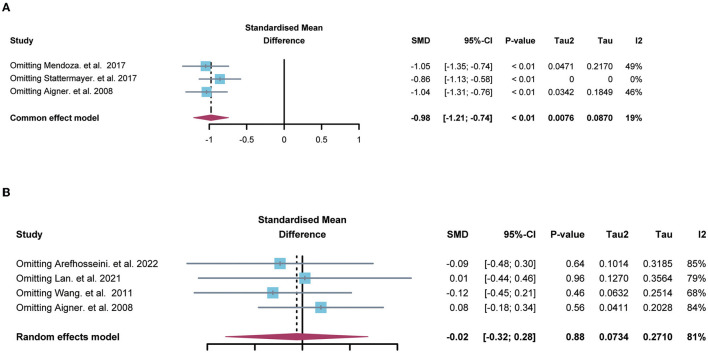
The forest plots showing the sensitive analysis of hepatic **(A)** and serum copper **(B)**. SMD, standardized mean differences; CI, confidence interval.

Sensitivity analysis suggested that no single study significantly influenced the difference on hepatic copper (Figure 3A) and serum copper (Figure 3B) in the comparison between NAFLD patients and normal individuals. Also, Egger's test indicated that there was no publication bias in the pooled estimates of hepatic copper (*p* = 0.73), and serum copper (*p* = 0.65). Given only 2 studies about serum ceruloplasmin, the sensitivity analysis and publication bias were not performed in the comparison of serum ceruloplasmin.

## Discussion

This meta-analysis revealed that hepatic copper concentration was significantly decreased in NAFLD patients and the sensitivity analysis also confirmed this. Nevertheless, serum copper and ceruloplasmin were not associated with NAFLD.

The total copper content in the adult body is 50–120 mg, and balancing the concentration of copper in the liver is essential for biological processes (20). It is reported that downregulation of hepatic ceruloplasmin results in the restoration of hepatic copper level, which attenuates liver steatosis by targeting copper-SOC1-AMPK signaling pathway in NAFLD mice model (20). What's more, copper inhibits the activation of PDE3B, a key enzyme of lipolysis, and the latter promotes c-AMP-dependent lipolysis (21). In addition, excessive copper induces lipogenesis and lipolysis through the activation of Nrf2-PPARγ pathway and autophagy (22). Moreover, copper induces cell death by targeting the lipoylated components of the tricarboxylic acid (TCA) cycle, which is called “cuproptosis” (9). Obviously, the homeostasis of copper plays an important role in lipid metabolism. Given the different results between liver copper and serum copper in our study, the biological function of liver copper and serum copper may be varying, and a positive correlation is found between serum copper and hepatic copper concentration in NAFLD patients, but there is no research contributing to the difference biological function between them (18).

Our research had the following advantages: (1) this study was the first to conduct a meta-analysis of the relationship between copper and NAFLD. However, our research also had some disadvantages: (1) the included studies were mainly case-control and cross-sectional study; (2) the meta-analysis of serum copper concentration still had substantial heterogeneity, and the removal of any literature did not improve this; (3) as few literatures on copper and NAFLD at present, few literatures were included; (4) the measurements used varied among the included studies, which increased the heterogeneity; (5) there was a strong possibility of sampling bias in our study. The liver copper cases were predominantly male (only 32% of subjects were female in the subset of studies with liver tissue data) which was not representative. Further, it was unclear what the severity of the disease was in the included studies (as an invasive test, people who were biopsied might have increased pre-test probability of more severe disease). According to Mendoza et al., advanced fibrosis may increase hepatic copper concentrations, which was part of the reason why that study excluded patients with high liver tissue copper (19); (6) we were unable to address the competing etiologies of copper deficiency and excess; (7) in the liver tissue studies, there are few controls for comparison, in part because of the invasive biopsy procedure required.

Therefore, this study suggests that the hepatic copper concentration is significantly decreased in NAFLD patients, and no difference is found in serum copper and ceruloplasmin between NAFLD patients and the normal individuals. Larger cohort studies and related trials are needed to further validate the result of this meta-analysis in the future.

## Author contributions

YC and CW contributed equally to this paper. YC, CW, and GL were involved with the study concept and design, acquisition of data, analysis, interpretation of data, drafting of the manuscript, and critical revision of the manuscript for important intellectual content. WW was involved in the acquisition of data. ST supervised the research and edited the manuscript. All authors contributed to the article and approved the submitted version.
